# Developing a measure of interpretation bias for depressed mood: An ambiguous scenarios test

**DOI:** 10.1016/j.paid.2011.04.005

**Published:** 2011-08

**Authors:** Chantal Berna, Tamara J. Lang, Guy M. Goodwin, Emily A. Holmes

**Affiliations:** Department of Psychiatry, University of Oxford, UK

**Keywords:** Depressed mood, Dysphoria, Cognitive bias, Interpretation bias, Cognitive bias modification, Mental imagery

## Abstract

The tendency to interpret ambiguous everyday situations in a relatively negative manner (negative interpretation bias) is central to cognitive models of depression. Limited tools are available to measure this bias, either experimentally or in the clinic. This study aimed to develop a pragmatic interpretation bias measure using an ambiguous scenarios test relevant to depressed mood (the AST-D).^1^ In Study 1, after a pilot phase (*N* = 53), the AST-D was presented via a web-based survey (*N* = 208). Participants imagined and rated each AST-D ambiguous scenario. As predicted, higher dysphoric mood was associated with lower pleasantness ratings (more negative bias), independent of mental imagery measures. In Study 2, self-report ratings were compared with objective ratings of participants’ imagined outcomes of the ambiguous scenarios (*N* = 41). Data were collected in the experimental context of a functional Magnetic Resonance Imaging scanner. Consistent with subjective bias scores, independent judges rated more sentences as negatively valenced for the high versus low dysphoric group. Overall, results suggest the potential utility of the AST-D in assessing interpretation bias associated with depressed mood.

## 1 Introduction

Biased information processing is an important marker of negative mood, and contributes to the development of depression and anxiety disorders (Mathews & Macleod, 2005). A negative interpretation bias refers to the attribution of a negative compared to a benign or positive meaning to an ambiguous situation (Butler & Mathews, 1983); it is relative, and considering a lack of positive interpretation bias is also of interest. Negative interpretation bias has been associated with clinical depression and depressed mood (dysphoria) (Butler & Mathews, 1983; Lawson, MacLeod, & Hammond, 2002; Rude, Valdez, Odom, & Ebrahimi, 2003). Cognitive models of depression suggest that negative interpretation bias – seeing one’s glass as perpetually half empty rather than half full – is critical to the maintenance of depressed mood (Beck, 1976). Promoting a less negative interpretation bias is an important component of successful cognitive behavioral therapy (CBT) for depression (Hollon et al. 2005).

CBM^2^ techniques have recently been developed to target such negative biases directly via computer-based training rather than face-to-face therapy (MacLeod, Koster, & Fox, 2009). For positive CBM interpretation bias (CBM-I), participants are trained to resolve situations that initially appear ambiguous in a benign/positive rather than negative way. CBM-I was initially developed in the context of anxiety disorders (e.g. Grey & Mathews, 2000; Mathews & Mackintosh, 2000). A CBM-I procedure emphasising the use of mental imagery to simulate scenarios, has been developed to reduce vulnerability to depressed mood (Blackwell & Holmes, 2010; Holmes, Lang, & Shah, 2009).

Experiments and treatments designed to modify interpretation bias would clearly benefit from tools to measure it. Perhaps surprisingly, the choice is currently limited; the measures include a physiological test – measuring the magnitude of blink reflex (from a puff of air to the eye) in response to ambiguous stimuli (Lawson et al. 2002) – and a behavioral test such as the Scrambled Sentences Task (Wenzlaff, Wegner, & Pennebaker, 1993). In the latter, participants are asked to make a sentence from a mixed sequence of words (under a cognitive load and constrained time). The words allow for ‘unscrambling’ into either a negative or positive sentence, thus providing an indication of bias. Such interpretation bias tests are not easy to administer and score. Other variants have not been submitted to basic scrutiny to determine whether they assess a bias relevant to dysphoria or depression (MacLeod et al. 2009). A more pragmatic measure for future use in clinical settings includes an ambiguous scenarios test (AST) in which participants are simply required to rate a series of descriptions (e.g. Holmes & Mathews, 2005; Holmes, Mathews, Dalgleish, & Mackintosh, 2006; Hoppitt, Mathews, Yiend, & Mackintosh, 2010). The initial version of the AST used recognition ratings and required a somewhat complex computation of a bias score (Mathews & Mackintosh, 2000). Replacing the recognition task with pleasantness ratings on a 9-point Likert scale simplified this (Holmes & Mathews, 2005). Further, to maximise impact, participants were encouraged to simulate the scenarios using mental imagery to resolve ambiguity (Holmes, Lang, & Shah 2009; Hoppitt et al. 2010). For example, one item read “You are watching the lottery results on TV. As the numbers are called you find out your result”. A positive interpretation would include winning and a negative interpretation, losing. Higher pleasantness ratings indicate a more positive interpretation bias. Since ASTs were initially developed for anxiety such a measure required modification to be valid in the context of depressed mood.

Our goal in the two studies presented here was to develop an AST measure of interpretation bias by adapting the scenario content for depressed mood (AST-D^1^). In line with Holmes, Lang, & Shah (2009), explicit instructions to imagine the ambiguous situations were included. We predicted that compared to low dysphorics (i.e. people with low levels of depressed mood), high dysphorics (people with high levels of depressed mood) would have a more negative bias on the AST-D (Study 1) as indicated by lower subjective pleasantness ratings. Further, we predicted that participants’ subjective ratings would be corroborated by independent raters’ judgments of written descriptions of the imagined scenarios (Study 2).

## 2 Study 1: Comparison of the ambiguous scenarios test in high versus low dysphorics

### 2.1 Overview

A 24-item AST-D was derived from a brief pilot study of 55 scenarios (*N *= 53). The AST-D was then presented in a web-based format (*N *= 208). Participants were instructed to imagine the outcome of each of the ambiguous scenarios, and to rate the pleasantness for each. To check whether differences in imagination were influencing the results, measures of mental imagery (vividness for the AST-D items and the tendency to use mental imagery in everyday life) were included. We predicted that the pleasantness scores on the AST-D would be negatively correlated with Beck Depression Inventory (BDI-II; Beck, Steer, & Brown, 1996) scores independent of the mental imagery measures.

## 3 Method

### 3.1 Pilot phase: AST-D item development

A pilot set of 55 items was derived from the 20-item AST used previously by Holmes and Mathews (2005), and Holmes et al. (2006) by adding 35 further depression-relevant items. People with clinical and research experience in depression devised the items. An example of a new scenario was “You wake up, get out of bed, stretch and really notice how you feel today.” This item could be interpreted either positively (e.g. they imagine feeling energetic), or negatively (e.g. they imagine feeling lethargic). A pilot study presented online these 55 scenarios to 53 participants (30 females, 78% aged between 18–34) whose BDI-II scores were recorded simultaneously. The participants with the 25% highest BDI-II (*M *= 14.75, *SD *= 4.39) and 25% lowest BDI-II (*M *= 0.33, *SD *= 0.65) scores were identified. For each scenario, the mean pleasantness ratings of the two groups were compared, choosing the 24 items showing largest effects. Thus, piloting reduced the initial 55-item set to 24 items, forming the AST-D (Appendix A) used in the current study.

### 3.2 Participants

E-mail invitations to university students allowed us to recruit 208 participants (136 females; mean age = 22.49 years, *SD *= 5.02). Participants had the opportunity to enter a cash prize draw of £100. This study, complying with the Ethical Recommendations of the British Psychological Society for online studies, received approval from the University of Oxford ethical board.

Two groups were generated according to the participants’ scores on the Beck Depression Inventory BDI-II Cut-offs of BDI-II ⩾ 14 (high dysphorics, *N *= 70) and of BDI-II ⩽ 6 (low dysphorics, *N *= 74) were chosen in line with previous research in this area (e.g. Holmes, Lang, Moulds, & Steele, 2008; Moulds & Kandris, 2006).

### 3.3 Procedure

Bristol Online Survey (2007) software was used to create the web-based survey. Participants gave informed consent online before beginning the questionnaires.

### 3.4 Measures

#### 3.4.1 AST-D

The 24 ambiguous scenarios were presented individually, followed by ratings e.g. “It’s New Year’s Eve. You think about the year ahead of you” (Appendix A). Participants were instructed to: “Form a mental image of each of the scenarios. Imagine each scenario happening to you personally. Follow the first image that comes to mind, don’t think too much about each one. Then rate how pleasant your image is, as well as how vivid it is.” The pleasantness rating was given on a 9-point Likert scale anchored from *extremely unpleasant* to *extremely pleasant*. The vividness rating was made on a 7-point Likert scale anchored from *not vivid at all* to *extremely vivid*. While the term ‘pleasantness rating’ is used henceforth, it does not simply refer to a ‘pleasant meaning positive’ dimension since its range extends from negative (extremely unpleasant) to positive (extremely pleasant).

#### 3.4.2 Spontaneous use of Imagery scale (SUIS;Reisberg, Pearson, & Kosslyn, 2003)*.*

The SUIS is a 12-item measure of the tendency to use imagery in everyday situations. Each item (e.g. “When I think about a series of errands I must do, I visualize the stores I will visit”) is rated on a 5-point Likert scale anchored at each point from (1) “*the description is… never appropriate”* to (5) “… *always completely appropriate”.*

BDI-II (Beck et al. 1996)*.* The BDI-II is a 21-item self-report questionnaire assessing symptoms of depression with good psychometric properties: the internal consistency is .93 with college students and .92 with psychiatric outpatients.

## 4 Results

### 4.1 Psychometric properties of the AST-D

Internal consistency was examined through reliability analysis. The AST-D had a Cronbach’s α of .82, indicating a good level of internal consistency (Barker, Pistrang, & Eliott, 1996). The corrected item-total correlations had a mean of .37. The pleasantness ratings of the scenarios were normally distributed (Shapiro–Wilk test: *W *= 0.995, *p* = .78). There was no significant difference in pleasantness ratings between men and women, *t*(206) = 0.96, *p* = .34, and no significant correlation with age (*r_s_* = .03, *p* = .63).

### 4.2 Correlation between scenario pleasantness and mood

As predicted, participants’ pleasantness ratings correlated negatively and significantly with their BDI-II score, *r*(206) = −.48, *p* < .001. Thus increased dysphoria was associated with a more negative interpretation bias. Partial correlations showed that when controlling for SUIS, the correlation remained significant *r*(205) = −.47, *p* < .001, as when controlling for AST-D vividness *r*(205) = −.51, *p* < .001. The range of BDI-II scores was 0–54.

### 4.3 A Comparison of high versus low dysphorics

The high and low dysphoric groups did not differ significantly in age, *t*(142) = 1.29, *p* = .20 or gender, *χ^2^*(1, *N *= 144) = 2.51, *p* = .11, see Table 1.

AST-D pleasantness ratings were compared between high and low dysphoric groups using independent samples *t*-tests. As predicted, the low dysphoric group rated the scenarios as significantly less pleasant than the high dysphoric group, *t*(142) = 5.95, *p* < .001, *d = *0.99, suggesting a more negative interpretation bias.

The vividness ratings for the AST-D items were not significantly different between the two groups, *t*(142) = 0.32, *p* = .75*.* The high dysphoric group reported greater spontaneous use of mental imagery in everyday life, as measured by the SUIS, *t*(142) = 2.83, *p* = .005, *d = *0.46.

## 5 Discussion

These results provide initial support for a simple to administer AST-D as an index of interpretation bias in depressed mood. Using a web-based study, the AST-D demonstrated good consistency in a population of students. The pleasantness ratings on this measure were negatively correlated with depressed mood (BDI-II), as would be predicted by the presence of a negative interpretation bias. This correlation was independent of vividness of the imagination of the AST-D scenarios, and of tendency to use mental imagery. Further, as predicted, high and low dysphoric groups differed significantly on the AST-D pleasantness ratings.

Although not key to our hypotheses, one unexpected finding was the higher SUIS scores in the high dysphoric group (Table 1). It is possible that such scores might reflect the presence of intrusive negative imagery – a feature of increasing research interest (Patel et al. 2007; Williams & Moulds, 2007). However, the mean values show only a modest difference and future research is needed to test replicability and further hypotheses about imagery in depression (Holmes , Lang & Deeprose, 2009).

It could be argued that since all ratings were subjective, the results may merely represent an anhedonic response. That is, dysphoric participants might subjectively experience less positive emotion in response to the imagery, rather than producing a more negative interpretation of the ambiguous stimuli per se. Participants’ actual interpretations were not recorded in this web-based study. Study 2 meant to address this issue by eliciting written descriptions of ambiguous scenarios’ imagined outcomes and using independent judges to rate these.

### 5.1 Study 2: Independent assessment of the resolved AST-D scenario descriptions in an fMRI^3^ setting

#### 5.1.1 Overview

Written descriptions of the ambiguous scenarios’ imagined outcomes were elicited so that interpretation bias could be rated both subjectively (as in Study 1) and by independent raters. The AST-D was presented in an experimental context – an fMRI scanning study, consistent with the aim to develop a tool to be used in a variety of settings. We predicted that the number of scenarios the judges rated negatively would correlate negatively with participants’ pleasantness ratings on the AST-D. Further, it was expected that more descriptions from high dysphoric participants would be objectively categorized as negative compared to descriptions from low dysphorics.

## 6 Method

### 6.1 Participants

Forty-one participants gave written informed consent (19 females, mean age 24.69 years, *SD *= 5.20). Participants were recruited through advertisements for an fMRI study on university mailing lists. The Oxfordshire Research Ethics Committee approved this study. Participants were divided into high and low dysphoric groups according to their scores on the *BDI-II*, as in Study 1.

### 6.2 Measures

BDI-II (Beck et al. 1996). The BDI-II served as a measure of depressed mood.

*AST-D.* In addition to giving pleasantness ratings (*measure of interpretation bias described in Study 1)*, participants described the scenarios’ imagined outcomes after coming out of the scanner. Vividness ratings were not included. Further details are given below.

### 6.3 Procedure

Participants were instructed to imagine the ambiguous scenarios as in Study 1. They were asked to remember each imagined outcome, in order to describe them once out of the scanner (technical limitations render this impossible during scanning). The scenarios were projected on a screen visible from the fMRI scanner (white characters, black background). Each scenario was split between two slides, the first presenting the context and the second containing the ambiguous outcome (e.g. “Slide 1: It’s New Year’s Eve. — Slide 2: You think about the year ahead of you.”). Slide 1 was displayed for 3–8s. according to the length of the text, slide 2 was always presented for 10s. allowing time to imagine the outcome. Participants also underwent a separate heat-perception task as part of a separate study described elsewhere (Berna, 2010).

After imagining each scenario, participants rated its pleasantness using a 2-button response device that moved a cursor continuously along a visual analogue scale presented on the screen, anchored from *extremely unpleasant* to *extremely pleasant*. After exiting the scanner, participants described each of the imagined outcomes on a sheet with the AST-D items.

Two independent raters (psychology researchers), blind to BDI-II scores, classified each of the 24 descriptions for each participant (*N *= 984 descriptions) into one of three categories: *negative*, *neutral/unclear* or *positive*. Inter-rater reliability between the first and the second judge was good (91%) (Barker et al. 1996). A third rater assessed cases of disagreement (*N *= 88). The majority answer was chosen. When all three raters disagreed, the scenario’s valence was considered *unclear* (*N *= 6). For each participant, a sum score of each of the negative, positive and neutral/unclear categories was computed separately.

## 7 Results

### 7.1 Subjective pleasantness ratings

As predicted, there was a significant negative correlation between depressed mood (BDI-II) and subjective pleasantness ratings, *r*(40) = −.56, *p* *<* .001. Further, compared to the low dysphoric group, the high dysphoric group rated their scenarios as less pleasant *t*(31) = 4.29, *p* = <.001, *d *= 1.6 (see Table 2).

### 7.2 Independent ratings

The mean number of descriptions in each valence category is shown in Table 2. Example resolutions for the item “It’s New Year’s Eve. You think about the year ahead of you” are: “It will be hard work, like this year, which I don’t look forward to” (negative valence); and “I’m excited and happy” (positive valence). BDI-II scores were significantly correlated with the number of scenarios the independent judges rated as positive, *r*(41) = −.63, *p* < .001, as well as with the number of scenarios rated as negative, *r*(41) = .53, *p* < .001, but not with the scenarios rated as neutral/unclear, *r*(41) = .17, *p *= 0.29.

The high dysphoric group’s scenarios were judged significantly more often as negative, *t*(31) = 3.29, *p* = .002, *d *= 1.24,, than those of the low dysphoric group, and significantly less often as positive, *t*(31) = 3.77, *p* = .001, *d *= 1.43, with no significant difference for the neutral category, *t*(31) = 0.77, *p* = .45.

### 7.3 Correlation between subjective and independent ratings

The subjective pleasantness ratings were significantly correlated with the objective ratings for the negative category, *r*(41) = −.60, *p *< 0.001, the positive category, *r*(41) = .70, *p *< 0.001, but not the neutral category *r*(41) = −.09, *p *= 0.59.

## 8 Discussion

Participants’ subjective AST-D ratings during fMRI scanning replicated findings from Study 1: depressed mood was associated with lower pleasantness ratings indicative of a more negative interpretation bias. Importantly, subjective and objective ratings showed good correspondence. Compared to those of low dysphorics, the descriptions of resolved ambiguous scenarios made by high dysphorics of resolved ambiguous scenarios were judged to be more often negative in content. This is consistent with the AST-D indexing a negative bias in interpretation rather than simply anhedonia.

## 9 General discussion

Our objective was a readily useable measure of interpretation bias relevant to dysphoria: the 24-item AST-D. In Study 1, the AST-D showed good internal consistency, correlated with depressed mood (BDI-II), and distinguished high versus low dysphorics. Findings were not explained by a lack of vividness in imagining the items or by a difference in tendency to use mental imagery in the high dysphoric group.

In Study 2, objective ratings confirmed that the descriptions of ambiguous scenarios imagined by a high compared to low dysphoric group were more negative in content. This is consistent with AST-D differences not merely being due to diminished positive affect for the same scenario outcome, but to differing interpretations of the outcome itself. Overall, the AST-D shows promise as a tool to assess interpretation biases for CBT treatment monitoring, experimental research such as CBM-I paradigms (e.g. Blackwell & Holmes, 2010) and during fMRI studies on similar topics (e.g. Browning, Holmes, Murphy, Goodwin, & Harmer, 2010).

These studies have a number of limitations. For example, they were conducted on non-clinical samples of students, and validating the AST-D in a general population as well as a clinical sample would be useful. In Study 2, time passed between the imagination and description of the scenarios. While this may have introduced extra variablity and weakened the results, a convergence between objective and subjective ratings was still found. Successful use of the AST-D in the environment of a MR scanner, suggests wide applicability. Finally, since some research suggests that lack of positivity bias is not the same as a negativity bias and there are different correlates, albeit in a different information processing framework (e.g. Hayden, Klein, Durbin, & Olino, 2006), further research might seek to develop versions of the AST-D, which could test this possibility.

Overall, results suggest the potential utility of the AST-D as a simple and thus pragmatic tool to assess interpretation bias associated with depressed mood. Depression and anxiety are highly comorbid and the relation between the two was beyond the scope of the current study but may be of interest in future studies. Since negative interpretation bias is central to cognitive models of depression, and measures are currently lacking both experimentally and in the clinic, the development of tools such as the AST-D is in high demand.

## Figures and Tables

**Table:** 

Your partner asks you to buy a present for their sister’s birthday, as they are busy. When the sister opens it, her face shows you how she feels.
Your best friend convinces you to go on a blind date and as you sit in the bar waiting to meet your date, you think about how it will go.
You give a speech at your friend’s wedding. When you have finished, you observe the audience’s reaction.
You wake up, get out of bed, stretch and really notice how you feel today.
You go to a place you visited as a child. Walking around makes you feel emotional.
You are about to move with your partner into a new home. You think about living there.
You are going to see your sister in her school play. You’ve left it to the last minute to get there. As you drive up to the school and see the parking bays you anticipate the time it will take you to arrive.
You are lost in a part of a big city you don’t know well. You ask someone on the streets for directions when they pull out something from their pocket.
You join a tennis club and before long you are asked to play in a doubles match. It’s a tough match and afterwards you discuss your performance with your partner.
You have recently taken an important exam. Your results arrive with an unexpected letter of explanation about your grade.
As you walk into the interview room the panel of interviewers welcomes you and proceeds to ask some tough questions. By the end of the interview you know what the outcome is.
You are starting a new job that you very much want. You think about what it will be like.
You go to a wedding where you know very few other guests. After the party, you reflect on how the other guests behaved.
You are organizing the annual office party on a small budget. On the night of the party, you look around to see if people are enjoying themselves.
You are going to see a very good friend at the station. You haven’t seen them for years. You feel emotional, thinking about how much they might have changed.
It’s New Year ’s Eve. You think about the year ahead of you.
You are in a reflective mood and think back at past achievements and disappointments that you have experienced during your life. Overall, your main feelings about your life so far emerge.
It is an overcast day and you are sitting on the beach. You look up to notice the weather really beginning to change.
Your neighbours have just had a new baby. You hear it crying. Through the window you see how the husband holds it.
You are camping in a forest and are very cold. You decide to light a fire. The flames grow in intensity much faster than you imagined.
Your friend is very keen on skating and persuades you to try it out. At the rink you put on the skates and step on the ice. You glide forward, slowly at first, then faster.
At the company you are working for there have been big cut backs. One day you are called into see your boss. When you enter the room, the boss’s face is tired.
You are interested in a job, but think you might be under-qualified and so ask for details. When you speak to the people, you realize what your chances are to get the job.
Some important people are visiting the office and you are asked at the last minute to present a project to them. Afterwards, you get feedback on your performance.

**Table 1:** Means and standard deviations for mood, bias and imagery scores of the high and low dysphoric groups in Study 1.

	High dysphoric (*N* = 70)		Low dysphoric (*N* = 74)	
	M	*SD*	M	*SD*
Age	21.67	4.98	22.77	5.22
Gender ratio (M:F)	17:53		27:47	
BDI-II	22.19	8.49	3.55	1.79
Interpretation bias – AST-D pleasantness ratings	5.03	0.76	5.84	0.86
Vividness of AST-D scenarios	4.74	0.87	4.70	0.81
SUIS	3.56	0.57	3.31	0.52

*Note*. BDI-II, Beck Depression Inventory-II; SUIS, Spontaneous Use of Imagery Scale; High dysphoric, BDI-II ⩾ 14; Low dysphoric, BDI-II ⩽ 6; AST-D, ambiguous scenarios test for dysphoria.

**Table 2:** Means and standard deviations for mood and bias scores (both subjective and objective) of the high and low dysphoric groups in Study 2.

	High dysphoric (*N* = 10)		Low dysphoric (*N* = 23)	
	M	SD	M	SD
Age	21.10	2.96	25.30	3.96
Gender ratio (M:F)	5:5		13:10	
BDI-II	22.70	4.40	2.74	2.30
AST-D pleasantness ratings (subjective)	4.84	0.60	5.75	0.56

*Independent ratings*				

Number of scenarios rated as				
Positive	6.70	3.09	11.09	3.06
Negative	10.70	3.16	6.83	3.08
Neutral/unclear	5.20	1.99	4.48	2.64

*Note.* BDI-II, Beck Depression Inventory-II; High dysphoric, BDI-II ⩾ 14; Low dysphoric, BDI-II ⩽ 6.

## Appendix A

The 24 ambigsuous scenarios of the AST-D

## Footnotes

2 Cognitive bias modification

3 Functional Magenetic Resonance Imaging
